# An evidence-based framework for postoperative surveillance of meningioma

**DOI:** 10.1093/nop/npae117

**Published:** 2024-12-02

**Authors:** Brittany Owusu-Adjei, Jeewoo C Lim, Connie C Hou, Constance J Mietus, Rrita Daci, William Lambert, Hanya Qureshi, Bethany C Berry, Madison R B Marasco, Umika Paul, Rachael W Sirianni, Mark D Johnson

**Affiliations:** UMass Memorial Health, Worcester, Massachusetts, US; Department of Neurological Surgery, University of Massachusetts Chan Medical School, Worcester, Massachusetts, US; UMass Memorial Health, Worcester, Massachusetts, US; Department of Neurological Surgery, University of Massachusetts Chan Medical School, Worcester, Massachusetts, US; Department of Neurological Surgery, University of Massachusetts Chan Medical School, Worcester, Massachusetts, US; UMass Memorial Health, Worcester, Massachusetts, US; Department of Neurological Surgery, University of Massachusetts Chan Medical School, Worcester, Massachusetts, US; UMass Memorial Health, Worcester, Massachusetts, US; Department of Neurological Surgery, University of Massachusetts Chan Medical School, Worcester, Massachusetts, US; UMass Memorial Health, Worcester, Massachusetts, US; Department of Neurological Surgery, University of Massachusetts Chan Medical School, Worcester, Massachusetts, US; UMass Memorial Health, Worcester, Massachusetts, US; Department of Neurological Surgery, University of Massachusetts Chan Medical School, Worcester, Massachusetts, US; Department of Neurological Surgery, University of Massachusetts Chan Medical School, Worcester, Massachusetts, US; Department of Neurological Surgery, University of Massachusetts Chan Medical School, Worcester, Massachusetts, US; Department of Neurological Surgery, University of Massachusetts Chan Medical School, Worcester, Massachusetts, US; Department of Neurological Surgery, University of Massachusetts Chan Medical School, Worcester, Massachusetts, US; UMass Memorial Health, Worcester, Massachusetts, US; Department of Neurological Surgery, University of Massachusetts Chan Medical School, Worcester, Massachusetts, US

**Keywords:** follow-up, meningioma, recommendations, recurrence, surveillance

## Abstract

**Background:**

Meningiomas frequently recur after surgery. Existing guidelines for postoperative surveillance are based on customary practices or limited data. This may result in excessive or inadequate surveillance.

**Methods:**

We compared 8 studies involving 1519 resected meningiomas with postoperative follow-up ranging from 7 to 23 years. Meningiomas were stratified using the World Health Organization and Simpson grading systems, and progression-free survival data were compared. Recurrence patterns were validated using 2 additional studies involving 2463 meningiomas.

**Results:**

Incompletely resected meningiomas of all grades displayed recurrences throughout the observation period. The 5-year and 10-year cumulative incidence of recurrence for completely resected Grade 1 meningiomas was 10% and 20%, with no recurrences beyond 11 years. For completely resected Grade 2 meningiomas, the 5-year and 10-year cumulative incidence of recurrence was 24% and 50%, with ongoing recurrences throughout the observation period. Elevated recurrence rates for Grade 1/2 meningiomas persisted beyond 5 years. For completely resected Grade 3 meningiomas, the 5-year cumulative incidence of recurrence was 63%, and all recurred before 10 years.

**Conclusions:**

Postoperative magnetic resonance imaging (MRI) at 48 h to determine the extent of resection and at 4 months to detect rapid regrowth is recommended. For completely resected Grade 1 meningiomas, annual MRI followed by discontinuation of surveillance if there is no recurrence after 11 years is reasonable. For completely resected Grade 2 meningiomas, annual MRI indefinitely is recommended. For Grade 3 meningiomas, MRI every 3-4 months for 2 years, followed by every 6 months indefinitely, is recommended. Incompletely resected meningiomas should be followed indefinitely.

**Key Points**

- Grade 1 meningiomas with GTR that have not recurred after 11 years may never recur.
- Grade 2/3 meningiomas with GTR recur frequently and should be followed indefinitely.
- Incompletely resected meningiomas require indefinite follow-up regardless of grade.

**Importance of the Study**

Existing guidelines for postoperative meningioma surveillance are based on physician experience, customary practices, or limited evidence from a small number of studies, with some offering the same recommendations for follow-up irrespective of tumor grade or extent of resection (EOR). This may lead to excessive or inadequate surveillance that compromises patient outcomes or unnecessarily increases costs. By comparing recurrence patterns for 3982 meningiomas, we confirm the influence of WHO grade and EOR on meningioma recurrence, report that recurrence rates for Grade 1 and 2 meningiomas remain largely unabated well beyond 5 years after surgery, and show that completely resected WHO Grade 1 meningiomas rarely recur beyond 11 years of observation. Based on these and other findings, we propose an evidence-based framework for meningioma surveillance aimed at optimizing the management and outcomes of meningioma patients while decreasing costs.

Meningioma is the most common intrinsic intracranial tumor, accounting for 39.7% of all intracranial tumors and 55.4% of all nonmalignant intracranial tumors.^1^ The mainstay of treatment for meningioma is surgical resection.^2^ In select cases, radiation provides an alternative to surgery or it can be used as an adjunct to surgical resection.^2,3^

The postoperative management of meningioma commonly involves prolonged follow-up with serial contrast-enhanced magnetic resonance imaging (MRI) to detect recurrence or the growth of residual disease. Numerous studies have examined progression-free survival (PFS) as a measure of meningioma recurrence.^4–7^ Most of these are single-center studies that have used different meningioma classification schemes, periods of follow-up, or standards for the inclusion of incompletely resected tumors in their analyses.^6,8–15^ Some multicenter studies have been reported, but these have not been used to develop evidence-based guidelines for meningioma surveillance. Consequently, the frequency and duration of radiological follow-up of meningiomas are often based on a mixture of customary practices and limited data. This creates considerable uncertainty and practice variation. Frequent or prolonged serial imaging for tumors that are unlikely to recur results in significant unnecessary costs and patient anxiety. Conversely, failure to image with an adequate frequency or duration of follow-up may delay the detection of recurrence and lead to worse outcomes.

The WHO grading system remains the most commonly used method for meningioma prognostication.^16^ It assigns a grade to each meningioma based on histological features with the goal of predicting the growth potential and risk of recurrence.^17–19^ WHO grade correlates with growth rate, recurrence risk, and overall survival.^17^ However, recent studies have found that the WHO grading system is not always prognostically accurate,^6^ as divergence in clinical course among meningiomas of the same WHO grade is not uncommon. Several investigators have proposed alternative molecular-based classification systems that better predict meningioma behavior.^6,10,13,20,21^ Although each of these molecular classification methods affords a more accurate prediction of the biological behavior of meningiomas than the WHO grading system, none are 100% accurate in their ability to predict meningioma recurrence, and none are in widespread clinical use. The low cost, accessibility, and demonstrated prognostic value of the WHO grading system have established it as the global standard of care for meningioma. Thus, to maximize their clinical utility at this time, evidence-based recommendations for postoperative meningioma surveillance may utilize the WHO meningioma classification system while simultaneously compensating for its failures in accurately predicting meningioma behavior.

Recurrence risk for meningioma is also affected by the extent of resection (EOR), which can be classified using the Simpson grading system.^22–24^ Complete resection is classified as Simpson Grade 1 (complete excision including associated dura and affected bone) or Simpson Grade 2 (complete excision with coagulation of dural attachments). Incomplete excision is classified as Simpson Grade 3 (complete excision without coagulation of dural attachments), Simpson Grade 4 (residual macroscopic disease), or Simpson Grade 5 (biopsy only).

The National Comprehensive Cancer Network (https://www.nccn.org/guidelines/category_1, NCCN) provides guidelines for postoperative meningioma surveillance.^25^ The 2023 NCCN guidelines provide the same recommendations for postoperative surveillance of WHO Grade 1 and Grade 2 meningiomas. However, these guidelines do not distinguish based on tumor grade or EOR. The NCCN recommendations for WHO Grade 1 and Grade 2 meningiomas include obtaining a brain MRI at 3, 6, and 12 months, followed by every 6-12 months for 5 years, and then every 1-3 years as clinically indicated. Although NCCN presents several references as the basis for these recommendations, the evidence that supports providing the same schedule of radiological follow-up for WHO Grade 1 and Grade 2 meningiomas regardless of the EOR, as well as the evidence supporting the recommendation to lengthen the interval of follow-up after 5 years of observation, is unclear. For WHO Grade 3 meningiomas, the NCCN recommends a brain MRI every 2-4 months for the first 3 years, followed by a brain MRI every 3-6 months thereafter. Importantly, NCCN guidelines do not address whether, after the complete removal of a meningioma, there is a time point at which a patient should be considered cured of their disease and no further surveillance is required.

Barriers to the development of an evidence-based framework for postoperative follow-up of meningioma include variations in how the WHO grading system is applied between different pathologists or institutions, inability of the WHO grading system to accurately predict the biological behavior of a minority of meningiomas, failure of studies to account for the EOR, low numbers of tumors analyzed in many studies, inadequate frequency or duration of follow-up, and potential differences in the biological behavior of these tumors based on differences in sex, race, or ethnicity of different patient populations.^19^ All of these factors make it difficult to rely on any single study to develop broadly applicable recommendations.

To overcome these barriers, we analyzed recurrence data for 1519 meningiomas obtained from 8 independent centers in North America, Europe, and Asia. Information on WHO grade, EOR, and duration of follow-up were incorporated into the analysis where available. Recurrence patterns for completely resected and incompletely resected meningiomas of all 3 WHO grades were compared across the 8 studies to identify common features. For validation, the results were compared with 2 additional studies involving 2463 meningiomas with information on WHO grade, EOR, and long-term follow-up.^23,24^ Taken together, this analysis of 3982 surgically resected meningiomas provides a broad-based view of meningioma recurrence that serves as a foundation for evidence-based recommendations regarding the appropriate frequency and duration of meningioma follow-up.

## Materials and Methods

**Figure 1.**
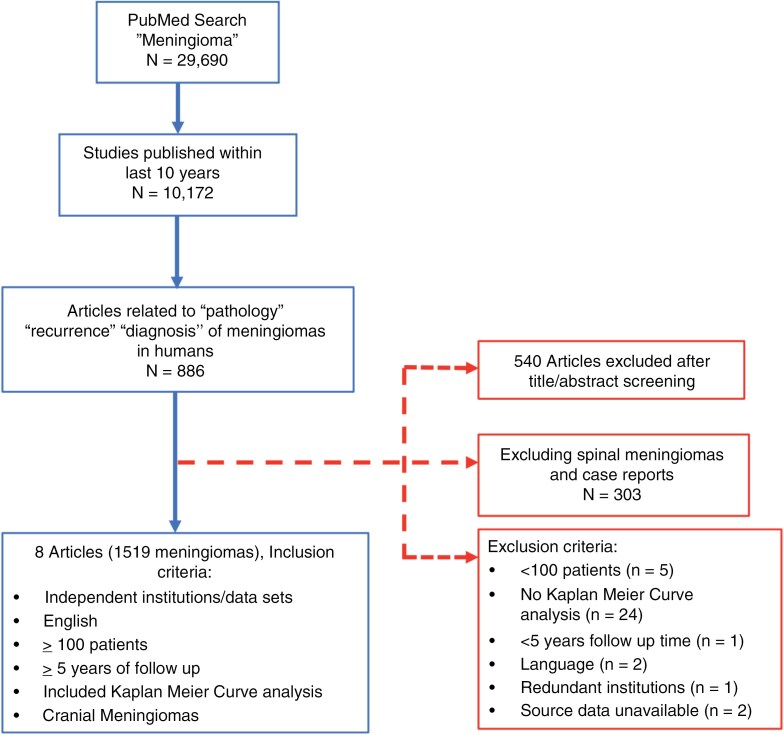
Flow diagram for selection of publications for comparative study of meningioma recurrence. A literature search in July 2023 using the PubMed database was performed with the retrieval of select articles on meningioma recurrence after applying inclusion and exclusion criteria.

A search of the literature was performed in July 2023 using the PubMed database with the following search terms: meningioma AND pathology AND recurrence AND diagnosis AND human; 886 articles were identified. Articles were excluded if they were case reports, studies on spinal meningiomas, or studies greater than 10 years old, resulting in 8 articles. The decision to exclude articles greater than 10 years old was made to ensure that studies used contrast-enhanced MRI (which became widely available at most major centers around the world by the early 1990s) to detect recurrence and included 1 to 2 decades of follow-up prior to publication.^26^ Studies were included that provided available source data on PFS for at least 100 meningiomas of different WHO grades that had more than 5 years of follow-up. Eight studies met these criteria and were included in the primary comparative analysis (Figure 1).

Data (including tumor grade, EOR, number of patients at risk, number of censored patients, and number and timing of recurrences) were extracted from Kaplan-Meier analyses for all 3 WHO meningioma grades. PFS data from Kaplan-Meier analyses for meningiomas of each WHO grade were comparatively analyzed and displayed using Graph Pad PRISM software. Censored patients were analyzed using the “right-censoring” method through Graph Pad PRISM software. Statistical significance was calculated using the log-rank (Mantel-Cox) test, with *P* < .05 being significant.

To obtain an integrated view of data from the various studies, PFS data from studies involving completely resected meningiomas were combined and analyzed separately for meningiomas of each WHO grade, and Kaplan-Meier analyses were then performed. An identical approach was used for studies where the EOR was not specified (and which presumably included a mixture of incompletely resected and completely resected tumors).

The findings obtained from comparing the 8 studies described above were compared with 2 additional studies involving 2463 meningiomas that included information on WHO grade, EOR, and long-term follow-up.^23,24^ Because the source data from these studies were not available, only qualitative comparisons using information derived from Kaplan-Meier PFS curves and the manuscript texts could be made.

## Results

Progression-free survival was evaluated for 1519 meningiomas (916 WHO Grade 1, 508 WHO Grade 2, 95 WHO Grade 3) across 8 independent studies from 5 countries (the United States, Germany, Norway, Canada, and Taiwan).^6,8–14^ All of the meningiomas underwent surgical resection. Six of the studies included meningiomas from all 3 WHO grades. One study only analyzed WHO Grade 1 and WHO Grade 2 meningiomas,^12^ and another study only analyzed WHO Grade 2 and WHO Grade 3 meningiomas (Supplementary Table 1).^14^ Studies either used the 2007 and/or 2016 WHO classification systems (Supplementary Table 1).

Four studies dealt exclusively with completely resected meningiomas (734 completely resected meningiomas in total, including 526 WHO Grade 1, 190 WHO Grade 2, and 18 WHO Grade 3 tumors).^6,9,11,12^ The remaining studies did not clearly specify the EOR in their analyses and were thus presumed to include a mixture of completely resected and incompletely resected meningiomas.^8,10,13,14^ Olar et al. separately analyzed completely resected and incompletely resected meningiomas and provided Simpson grades for each tumor, allowing for detailed analysis.^11^ Of note, gross total resection (GTR) was defined as Simpson Grade 1 or 2 in 2 studies^9,11^ and was based on the analysis of postoperative MRI in the other 2 studies.^6,12^ The radiation status of patients was variable among studies, with some including nonirradiated patients,^6,12^ others including patients receiving adjuvant postoperative radiation along with nonirradiated patients,^8,10,14^ and still others that did not report radiation status.^9,11,13^

### Recurrence Patterns for WHO Grade 1 Meningiomas

**Figure 2.**
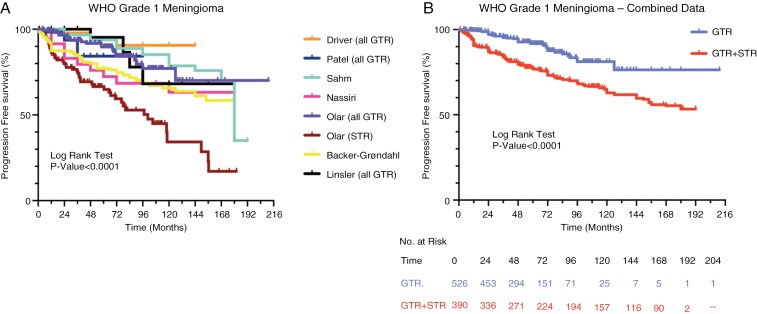
Comparative analysis of progression-free survival (PFS) for WHO Grade 1 meningioma. (A) Kaplan-Meier analyses from 7 separate studies evaluating PFS of patients with WHO Grade 1 meningiomas. Studies that exclusively analyzed meningiomas that underwent complete/gross total resection (GTR) are indicated. Data from Olar et al. was separated into a group of meningiomas undergoing GTR or a group of meningiomas undergoing subtotal resection (STR) to assess the effects of EOR on PFS. (B) Kaplan-Meier analysis using combined data from 4 studies that evaluated PFS for WHO Grade 1 meningiomas underwent GTR. A separate Kaplan-Meier analysis utilized data from 4 studies where WHO Grade 1 meningiomas that underwent either GTR or STR were considered together (GTR + STR). The log-rank test was used to calculate statistical significance.

WHO Grade 1 meningiomas (*n* = 916)^6,8–13^ were analyzed in 7 studies, 4 of which included only completely resected tumors (*n* = 526).^6,9,11,12^ Data from Kaplan-Meier PFS analyses from these studies were plotted together for comparison (Figure 2A). Across 6 of the studies^6,8–12^ (excluding partially resected cases in Olar et al.^11^), more than 60% of patients were recurrence free by the end of the individual observation periods. Combining the data from the 4 studies that separately evaluated the recurrence of completely resected tumors revealed that the median PFS for completely resected WHO Grade 1 meningiomas was not reached after over 17 years of observation. The average cumulative incidence of recurrence for completely resected WHO Grade 1 meningiomas at 5, 10, and 15 years was 10%, 20%, and 24%, respectively. The rate of recurrence remained steady until about 8 years postoperatively, at which time it began to decrease. Importantly, no recurrences were observed after 11 years of follow-up for completely resected WHO Grade 1 meningiomas, despite continuing the observation period for an additional 6 years beyond the 11-year time point.

When a mixture of incompletely resected and completely resected WHO Grade 1 meningiomas was analyzed, a significant decrease in median PFS was observed when compared with that of completely resected WHO Grade 1 meningiomas (*P* < .0001, hazard ratio (HR) 0.41, 95% confidence interval (CI) 0.30-0.56) (Figure 2B). Over a 17-year period of observation, the cumulative incidence of recurrence for this mixture of incompletely resected and completely resected WHO Grade 1 meningiomas was 48% at 15 years. Recurrences were observed throughout the 17-year observation period.

### Recurrence Patterns for WHO Grade 2 Meningiomas

**Figure 3.**
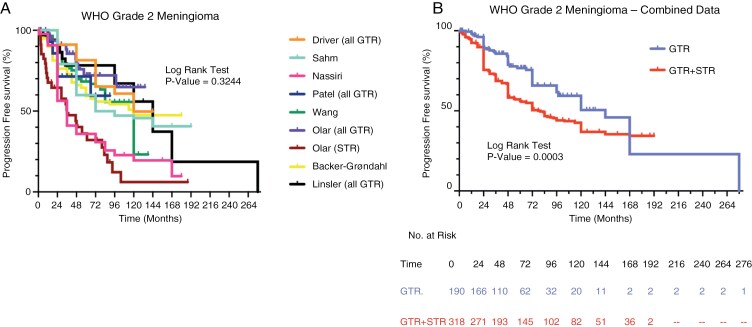
Comparative analysis of progression-free survival (PFS) for WHO Grade 2 meningioma. (A) Kaplan-Meier analyses from 8 separate studies evaluating PFS of patients with WHO Grade 2 meningiomas. Studies that exclusively analyzed meningiomas that underwent complete/gross total resection (GTR) are indicated. The remaining studies did not specify EOR and were presumed to include meningiomas that underwent GTR as well as meningiomas that underwent subtotal resection (GTR + STR). (B) Kaplan-Meier analysis of PFS using combined data from 4 studies examining WHO Grade 2 meningiomas that underwent GTR. A separate Kaplan-Meier analysis utilized data from 5 studies where WHO Grade 2 meningiomas that underwent either GTR or STR were considered together (GTR + STR). The log-rank test was used to calculate statistical significance.

WHO Grade 2 meningiomas (*n* = 508) were analyzed across 8 studies, each demonstrating variable estimates of median PFS (Figure 3A).^6,8–14^ For completely resected Grade 2 meningiomas, the median PFS was approximately 10-12 years. The cumulative incidence of recurrence for completely resected WHO Grade 2 meningiomas was 24% at 5 years, 50% at 10 years, and 78% at 15 years. Thus, a significant majority of completely resected Grade 2 meningiomas recurred within 15 years. The frequency of recurrences decreased noticeably after 12 years of observation but continued throughout the observation period.

Examination of data from a group that included a mixture of completely resected and incompletely resected WHO Grade 2 meningiomas (Figure 3B) revealed a decreased median PFS of approximately 6 years when compared with completely resected WHO Grade 2 meningiomas (*P* = .0003, HR 0.59, 95% CI 0.44-0.78). Importantly, 66% of these tumors recurred/regrew within 16 years. Although the rate of recurrence decreased after 10 years of observation, recurrence/regrowth events continued to occur throughout the 16-year observation period.

### Recurrence Patterns for WHO Grade 3 Meningiomas

**Figure 4.**
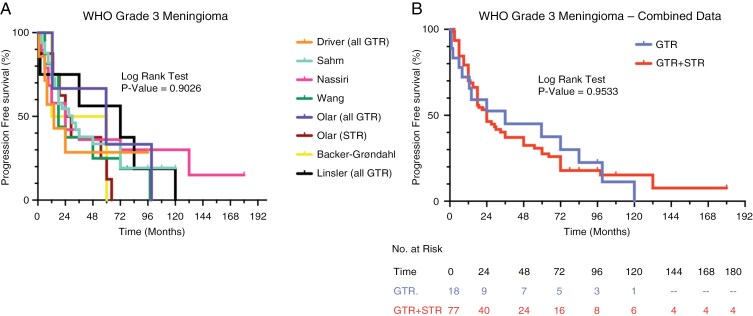
Comparative analysis of progression-free survival (PFS) for WHO Grade 3 meningioma. (A) Kaplan-Meier analyses from 7 separate studies evaluating PFS of patients with WHO Grade 3 meningiomas. Studies that exclusively analyzed meningiomas that underwent complete/gross total resection (GTR) are indicated. The remaining studies did not specify EOR and were presumed to include meningiomas that underwent GTR as well as meningiomas that underwent subtotal resection (GTR + STR). (B) Kaplan-Meier analysis of PFS using combined data from 3 studies examining WHO Grade 3 meningiomas that underwent GTR. A separate Kaplan-Meier analysis utilized data from 5 studies where WHO Grade 3 meningiomas that underwent either GTR or STR were considered together (GTR + STR). The log-rank test was used to calculate statistical significance.

Seven studies included an evaluation of recurrence patterns for WHO Grade 3 meningiomas (*n* = 95).^6,8–11,14^ Completely resected Grade 3 meningiomas had a median PFS of 3 years, which was the shortest among the different WHO grades of meningioma (Figure 4A). Importantly, there was no statistically significant difference in PFS between the group of completely resected WHO Grade 3 meningiomas and a group containing a mixture of completely resected and partially resected WHO Grade 3 meningiomas (24 months vs. 36 months, Figure 4B, *P* = .9533, HR 1.02, 95% CI 0.55-1.87). The cumulative incidence of recurrence at 5 years for completely resected WHO Grade 3 meningiomas was 62%, and all of the tumors in this cohort of 18 completely resected WHO Grade 3 meningiomas had recurred by 10 years. Recurrences were observed as early as 1 month postoperatively and continued throughout the observation period.

For incompletely resected Grade 3 meningiomas, the cumulative incidence of recurrence at 5, 10, and 15 years was 73%, 86%, and 93%, respectively. The median PFS for this group was 2 years. The rate of recurrence for both completely resected and incompletely resected Grade 3 meningiomas was especially high during the first 2 years after surgery, and it decreased somewhat thereafter.

### Comparison to an Independent Meningioma Cohort

To confirm the generalizability of the findings obtained here and their suitability for establishing a general framework for postoperative meningioma surveillance, we identified 2 additional studies^23,24^ that took WHO grade and the EOR into account when examining PFS after meningioma surgery. These studies were not integrated into our primary comparative analysis because the authors combined WHO Grade 2 and Grade 3 tumors together in Kaplan-Meier analyses where the extent of tumor resection was considered,^24^ or because the source data (including numbers of censored patients or patients at risk) needed to incorporate these studies into the primary comparative analysis were not available. However, both studies provided Kaplan-Meier PFS curves that served as useful comparators to our primary comparative analysis of meningioma recurrence patterns.

**Figure 5.**
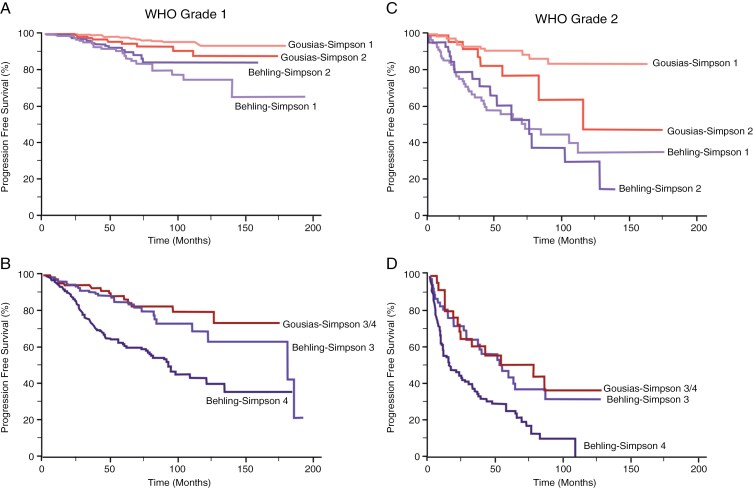
Validation comparative analysis of progression-free survival (PFS) for WHO Grade 1 and WHO Grade 2 meningiomas. (A) Kaplan-Meier analyses from 2 independent studies (Gousias et al. and Behling et al.) evaluating PFS of patients with WHO Grade 1 meningiomas that underwent complete resection (Simpson Grades 1 and 2). (B) Kaplan-Meier analyses from 2 independent studies (Gousias et al. and Behling et al.) evaluating PFS of patients with WHO Grade 1 meningiomas that underwent incomplete/subtotal resection (STR) (Simpson Grades 3 and 4). (C) Kaplan-Meier analyses from 2 independent studies (Gousias et al. and Behling et al.) evaluating PFS of patients with WHO Grade 2 meningiomas that underwent complete resection (Simpson Grades 1 and 2). Of note, Behling et al. included WHO Grade 3 meningiomas along with their analysis of WHO Grade 2 meningiomas. (D) Kaplan-Meier analyses from 2 studies (Gousias et al. and Behling et al.) evaluating PFS of patients with WHO Grade 2 meningiomas that underwent incomplete/STR (Simpson Grades 3 and 4). Of note, Behling et al. included WHO Grade 3 meningiomas along with their analysis of WHO Grade 2 meningiomas.

Behling et al. included an evaluation of recurrence patterns for 1571 meningiomas, including 1251 WHO Grade 1 meningiomas, 295 WHO Grade 2 meningiomas, and 25 WHO Grade 3 meningiomas.^24^ Inspection of the PFS curves for their cohort of 631 completely resected WHO Grade 1 meningiomas revealed that the median PFS was not reached after 15 years of observation. The cumulative incidence of recurrence at 10 years was about 23%, which is similar to the cumulative incidence of recurrence of 20% at 10 years observed in our primary comparative analysis of 526 completely resected WHO Grade 1 meningiomas. In the study by Behling et al., completely resected WHO Grade 1 meningiomas showed no recurrences after approximately 11.5 years of observation (Figure 5A).

A separate study by Gousias et al. examined recurrence patterns for 901 meningiomas, including 716 WHO Grade 1 meningiomas, 174 WHO Grade 2 meningiomas, and 11 WHO Grade 3 meningiomas.^23^ For completely resected WHO Grade 1 meningiomas, median PFS was not reached after 15 years of observation. There were no recurrences of completely resected WHO Grade 1 meningiomas after 10 years of observation (Figure 5A), supporting the findings of our primary comparative analysis that WHO Grade 1 meningiomas rarely recur beyond 11 years of observation.

In contrast to the findings for completely resected WHO Grade 1 meningiomas, analysis of 612 incompletely resected (Simpson Grades 3 and 4) WHO Grade 1 meningiomas by Behling et al. revealed recurrences more than 15 years after surgery, a pattern that was also observed in our primary comparative analysis of 8 independent studies.^24^ However, Gousias et al. saw no recurrences among incompletely resected Grade 1 meningiomas beyond 11 years of observation (Figure 5B).

Gousias et al. examined recurrence patterns for 174 WHO Grade 2 meningiomas and found that the median PFS was not reached after 10 years of observation. Interestingly, no recurrences of completely resected Grade 2 meningiomas were observed after 10 years in that study.^23^ In the study by Behling et al., the median PFS for a group containing 153 completely resected WHO Grade 2 and Grade 3 meningiomas was approximately 6 years (Figure 5C). The cumulative incidence of recurrence for this mixed group of meningiomas was about 65% at 10 years. Incompletely resected WHO Grade 2 and Grade 3 meningiomas recurred throughout the observation period (Figure 5D).^24^

A recent study by Chen et al.^20^ reported the development of a gene expression-based meningioma classification system that predicts prognosis and radiation response more accurately than the WHO classification system or several other previously reported molecular-based classification systems.^20^ They obtained 5-year PFS estimates of 96.1% for low-risk, 80.5% for intermediate-risk, and 30.0% for high-risk meningiomas that were completely resected.^20^ These PFS rates are comparable to the 5-year PFS rates of 91.7%, 76.7%, and 36.4% observed in the current study for completely resected WHO Grade 1, 2, and 3 meningiomas, respectively. Importantly, they observed no recurrences of completely resected low-risk meningiomas after 6 years, which may be compared with our finding of no recurrences after 11 years for completely resected WHO Grade 1 meningiomas. Similar to our findings for WHO Grade 2 meningiomas, Chen et al. observed ongoing recurrences throughout the 10-year observation period among completely resected intermediate-risk meningiomas.^20^

Chen et al. also evaluated a prospective meningioma cohort from the RTOG 0539 clinical trial.^20^ They reported 5-year PFS rates for low-, intermediate-, and high-risk meningioma subgroups of 92%, 76.5%, and 38.6%, respectively.^20^ These prospective data align closely with the PFS rates for completely resected WHO Grade 1 (91.7%), Grade 2 (76.7%), and Grade 3 (36.4%) meningiomas obtained in the current study. Together, these findings support the use of the widely available and cost-effective WHO classification system for the development of evidence-based recommendations for meningioma surveillance.

### An Evidence-Based Framework for Meningioma Surveillance

**Figure 6.**
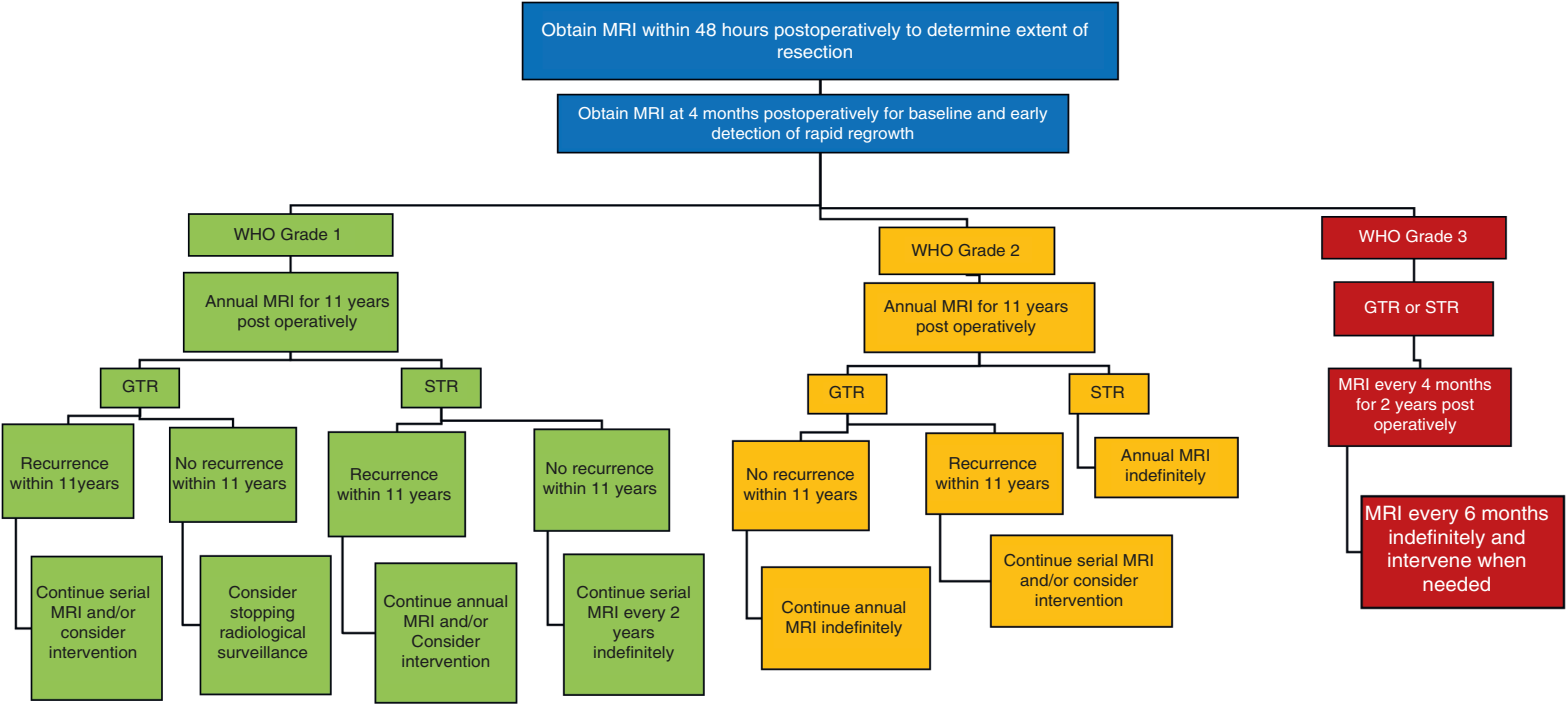
An evidence-based framework for postoperative meningioma surveillance.

Based on the patterns of meningioma recurrence identified in this comparative study, we have developed an evidence-based framework for postoperative radiological follow-up of meningiomas that takes into account WHO grade, EOR, and the observed time course of meningioma recurrence across several independent populations (Figure 6). Recommendations are made for initial short-interval postoperative imaging that will provide critical baseline data regarding the EOR and the demonstrated growth behavior of each tumor. This initial set of data will inform the recommended long-term schedule of follow-up for each patient.

A postoperative contrast-enhanced MRI should be obtained within 48 h after surgical resection of meningioma to determine the EOR and establish the size and location of gross residual disease. As reported in the literature, this early time point is chosen because it precedes the development of a robust postsurgical inflammatory response which causes surgically induced contrast enhancement (an effect that can last several days to weeks) that can be confused with residual tumor on contrast-enhanced MRI studies.^27–30^

To compensate for potential errors in prognostication arising from the use of the WHO grading system (eg misclassifying an aggressive meningioma as benign), we recommend that another short-interval contrast-enhanced MRI be obtained 4 months postoperatively. This early time point should detect rapidly growing tumors, and it has the added advantage of providing baseline images that are free of the potentially confounding acute postoperative changes that are commonly observed during the first few days and months after surgery. After the WHO grade of the meningioma has been determined by the pathologist, the EOR (ie the Simpson Grade) has been reported by the surgeon and confirmed by a contrast-enhanced MRI obtained within 48 h of surgery, and an MRI has been obtained after 4 months to rule out a gross underestimation of the growth rate by the assigned WHO grade, an evidence-based plan for subsequent radiological follow-up can be tailored for each meningioma patient.

For WHO Grade 1 meningiomas that have been completely resected, a contrast-enhanced MRI every year for the first 11 years postoperatively is recommended. If no recurrence is observed after 11 years of observation, the evidence presented here suggests that clinicians may consider discontinuing radiological surveillance because the risk of recurrence for completely resected WHO Grade 1 meningiomas beyond that time point is very low. Although the rate of recurrence/regrowth for WHO Grade 1 meningiomas that have undergone subtotal resection (STR) decreased after 11 years, episodes of recurrence/regrowth continued to occur. Thus, for incompletely resected WHO Grade 1 meningiomas, the evidence supports annual contrast-enhanced MRI for the first 11 years, followed by contrast-enhanced MRI studies every 2 years indefinitely if there has been no evidence of regrowth at the 11-year time point. This increase in the interval between follow-up MRI scans for residual WHO Grade 1 meningiomas that show no evidence of growth is made possible by the demonstrated low growth potential of these tumors over the preceding 11 years of observation.

For completely resected WHO Grade 2 meningiomas, an annual contrast-enhanced MRI is recommended for the first 11 years postoperatively, followed by imaging every year thereafter indefinitely if there is no evidence of recurrence. The recommendation to continue imaging every year despite the decreased frequency of recurrence after 11 years of observation is based upon the increased growth rate displayed by these tumors and the known tendency for some WHO Grade 2 meningiomas to become more aggressive and grow more rapidly over time.^5,17,31^ For incompletely resected WHO Grade 2 meningiomas, annual contrast-enhanced MRI indefinitely is recommended because of the increased rate of growth, potential for progression to a more aggressive phenotype, and enduring risk of regrowth associated with residual macroscopic disease.

The cumulative incidence of recurrence is high for WHO Grade 3 meningiomas and approaches 90% at 15 years, regardless of the EOR. Because of their rapid growth rate and extremely high risk of recurrence, appropriate surveillance of these tumors should involve short-interval follow-up for an extended period of time. The rate of recurrence for these tumors is highest during the first 2 years after surgery, although it moderates somewhat thereafter. We, therefore, recommend an MRI every 4 months for the first 2 years, followed by an MRI every 6 months indefinitely for radiological surveillance of WHO Grade 3 meningiomas, regardless of the EOR.

## Discussion

The evidence-based framework for postoperative meningioma surveillance proposed here is based on the comparative evaluation of PFS for 1519 meningiomas described in 8 studies from independent institutions scattered around the world. The findings from these 8 studies were compared with those from 2 additional studies involving 2463 surgically resected meningiomas. Thus, this study evaluated recurrence data for 3982 meningiomas in 10 studies and revealed patterns of meningioma recurrence that were remarkably similar across the different studies and patient populations. This framework for postoperative meningioma surveillance takes into account differences in EOR and the biological behavior of meningiomas of different WHO grades. In addition, the proposed framework accounts for the occasional designation of rapidly growing tumors as WHO Grade 1 meningiomas by incorporating a short-interval MRI early in the surveillance protocol to detect rapidly growing tumors at an early stage.

We recommend annual evaluations (as opposed to twice each year or every 2 years) for long-term surveillance of WHO Grade 1 and Grade 2 meningiomas for 2 reasons. First, recurrent meningiomas are often more aggressive than the initial tumor, and the shorter follow-up interval of 1 year provides increased patient safety. Conversely, most Grade 1 meningiomas grow by less than 2 cm^3^/year,^32^ so that imaging at intervals less than 1 year is unlikely to detect actionable growth. Second, early detection while the recurrence is small minimizes the risk of symptom development and maintains the option of stereotactic radiosurgery as an alternative to surgery.

In evaluating evidence to support the development of a framework for postoperative meningioma surveillance, we chose to perform a comparative analysis rather than a meta-analysis because of the heterogeneity among the studies included here. The comparative analysis provides a side-by-side comparison of the results of each study that makes similarities and differences between them apparent. Despite differences in methodology among these studies, similar patterns of meningioma recurrence were observed, and these patterns served as the basis for the evidence-based framework proposed here.

In the 2023 NCCN guidelines for meningioma surveillance, WHO Grade 1 and Grade 2 meningiomas share the same postoperative recommendations. Likewise, EOR is not used to develop distinct surveillance schedules for these tumors. The finding that meningioma recurrence patterns are strongly influenced by WHO grade and Simpson grade provides a strong rationale for developing postoperative surveillance plans that take these parameters into account. In addition, current NCCN guidelines for postoperative surveillance of WHO Grade 1 and Grade 2 meningiomas recommend that if there has been no evidence of recurrence after 5 years of observation, the interval for follow-up may be increased from once every year to once every 2-3 years. However, we observed that the rates of recurrence observed in the early years of postoperative surveillance of WHO Grade 1 and Grade 2 meningiomas continue unabated well beyond 5 years after surgery. For completely resected WHO Grade 1 meningiomas, these rates decreased between 8 and 11 years after surgery, whereas for Grade 2 meningiomas this occurred between 10 and 14 years after surgery. Thus, the evidence supports a surveillance schedule that extends the annual imaging period for Grade 1 and Grade 2 meningiomas to 10 years or more after surgery before lengthening the follow-up interval.

One factor that was not considered in the current study is whether adjuvant radiation was administered after surgical resection. Although many studies have examined the effects of radiation on meningioma recurrence, the use and long-term benefits of radiation for Grade 1 and Grade 2 meningiomas remain controversial, with some studies reporting a benefit and others not.^3,20,33–35^ Two phase III randomized prospective trials (NRG-BN-003 and ROAM/EORTC 1308) comparing surgery plus adjuvant radiation to surgery alone for WHO Grade 2 meningiomas will help to address this question.^36,37^ In most centers, adjuvant radiation is routinely administered after surgery for Grade 3 meningiomas because of the high risk of recurrence. Because many aggressive meningiomas are resistant to radiation, and because the inhibitory effect of radiation on tumor growth can be time-limited, the need for close surveillance of aggressive meningiomas over a long period of time remains. Importantly, the gene expression-based approach of Chen et al. had an AUC of 0.81. It was not capable of conclusively predicting which individual meningiomas would recur, and both favorable and unfavorable meningiomas showed extensive recurrence after radiation.^20^ Thus, tumor surveillance is required after surgery, regardless of the EOR, the use of adjuvant radiation, or the meningioma classification system used. Some of the studies included in our analysis likely included meningiomas that received postoperative irradiation, but these data were not clearly presented in their publications. Thus, the framework for postoperative meningioma surveillance presented here relies on data that incorporate the purported benefits of adjuvant irradiation for meningioma based on how it is typically utilized to treat meningiomas in clinical practice.

This comparative analysis is limited by the fact that the studies included were retrospective and did not describe which lesions were symptomatic or recurrent. As a result, the duration of postoperative surveillance varied among studies, and the analysis was affected by patients who were lost to follow-up. Because of the slowly growing nature of meningiomas, a follow-up of 5-15 years or more is necessary to understand the long-term risk of recurrence after all macroscopic diseases and their attachments have been removed. These limitations were partially mitigated by comparing numerous studies (some with follow-up as long as 23 years). The possibility that a completely resected Grade 1 meningioma can be cured is a real one, given that a majority of such tumors fail to recur after decades of radiological follow-up. Because our evidence-based framework for postoperative meningioma surveillance responds to this reality by suggesting that radiological follow-up of completely resected Grade 1 meningiomas may be discontinued after 11 years of follow-up with no recurrence, it has the potential to decrease patient anxiety, inconvenience, and costs.

The findings of our analysis are limited by inconsistencies in how the EOR was reported among the studies evaluated. Whereas several of the studies solely analyzed meningiomas that had undergone GTR, others did not specify the EOR and presumably grouped meningiomas that had undergone GTR or STR together for analysis. In our primary comparative analysis, we could readily compare recurrence-free patterns for completely resected meningiomas of different WHO grades across multiple studies. Our primary multi-study comparison also enabled us to compare data from completely resected tumors with data from a mixture of completely resected and incompletely resected meningiomas. However, only one study in our primary analysis (Olar et al.) directly compared recurrence patterns for completely resected meningiomas with recurrence patterns for incompletely resected tumors. Fortunately, the studies by Gousias et al. and Behling et al.,^23,24^ which were used to validate the findings of the primary analysis, also compared PFS for completely resected meningiomas of different WHO grades with that of incompletely resected meningiomas. All 3 studies revealed shorter PFS times and higher rates of recurrence/regrowth for incompletely resected meningiomas when compared with completely resected meningiomas or groups containing a mixture of completely resected and incompletely resected tumors.

The framework for postoperative meningioma surveillance proposed here is based on the WHO grading system for meningiomas, which remains the standard of care around the world at the time that this manuscript was written. However, molecular-based classification systems for meningioma yield more accurate predictions of the growth potential and recurrence risk of these tumors. New proposed molecular-based classification systems for meningioma require different levels of technological or statistical expertise that affect their accessibility. As one or more of these molecular classification systems is more widely adopted, the evidence-based recommendations for meningioma surveillance proposed here may need to be modified to incorporate these more accurate molecular classification systems. Notably, a comparison of the recently reported gene expression-based classification system of Chen et al.^20^ with the WHO classification system for meningioma indicated that the aggregate patterns of recurrence for low-, intermediate-, and high-risk meningiomas are very similar to those observed for WHO Grade 1, 2, and 3 meningiomas. Thus, the evidence-based surveillance framework provided here, which is based on the widely used WHO classification system, is currently appropriate and accessible for use in a wide range of clinical settings. Likewise, any of the recently published molecular meningioma classification schemes could be used with this surveillance framework as well.^38^

## Supplementary material

Supplementary material is available online at *Neuro-Oncology Practice* (https://academic.oup.com/nop/).

npae117_suppl_Supplementary_Table
