# Supplementary material for: An evidence-based framework for postoperative surveillance of meningioma
Source: Neurooncol Pract. 2024 Dec 2;12(3):478–88. doi: 10.1093/nop/npae117 (PMC12137216; doi:10.1093/nop/npae117)
Supplement: npae117_suppl_Supplementary_Table [file npae117_suppl_supplementary_table.docx]

| **Author and Year** | **Country** | **WHO Classification Year** | **WHO Grade (N)** | **Resection** | **Previous Radiation** |
| --- | --- | --- | --- | --- | --- |
| Driver et al., 2022^6^ | United States | 2007 & 2016 | Grade 1 - 240  Grade 2 - 91  Grade 3 - 7 | All GTR ^a^ | None |
| Nassiri et al., 2021^10^ | Canada | 2016 | Grade 1 - 59  Grade 2 - 43  Grade 3 - 19 | Variable ^b^ | Variable |
| Patel et al., 2019^12^ | United States | 2016 | Grade 1 - 61  Grade 2 - 14 | All GTR ^a^ | None |
| Sahm et al., 2017^13^ | Germany | 2016 | Grade 1 - 91  Grade 2 - 96  Grade 3 - 32 | Variable ^b^ | Unknown |
| Wang et al., 2016^14^ | Taiwan | 2007 | Grade 2 - 86  Grade 3 - 16 | Variable ^b^ | Variable |
| Backer-Grøndahl et al., 2014^8^ | Norway | 2007 | Grade 1 - 135  Grade 2 - 59  Grade 3 - 2 | Variable ^b^ | Variable |
| Linsler et al., 2014^9^ | Germany | 2007 | Grade 1 - 62  Grade 2 - 35  Grade 3 - 8 | All GTR ^c^ | Unknown |
| Olar et al., 2014^11^ | United States | 2007 | Grade 1 - 268  Grade 2 - 84  Grade 3 - 11 | Both GTR ^c^ and STR | Unknown |
|  | Abbreviations: GTR (gross total resection), STR (subtotal resection) | | | | |
|  | ^a^ Extent of resection based on imaging  ^b^ GTR and STR not distinguished within the cohort  ^c^ Simpson Grade 1 or 2 | | | | |

**Supplementary Table 1: Studies included in primary comparative analysis of progression free survival for meningioma**
